# Development of Acid-Sensitive Platinum(II) Complexes With Protein-Binding Properties

**DOI:** 10.1155/MBD.2000.89

**Published:** 2000

**Authors:** M. T. Schütte, R. Mülhaupt, F. Kratz

**Affiliations:** 1 Institute of Macromolecular Chemistry University of Freiburg Stefan-Meier-Straße 31 Freiburg 79104 Germany; 2 Tumor Biology Center Department of Medical Oncology Clinical Research Breisacher Straße 117 Freiburg 79106 Germany

## Abstract

Four new protein-binding platinum(II) complexes, **10**, **11**, **21**, **22**, in which the dichloroplatinum moiety is coordinated either to a carbon-substituted or a nitrogen-substituted ethylene diamino ligand, were prepared in ten-step syntheses. According to pH-dependent stability studies with strictly related compounds, **11** and **22** exhibit acid-sensitive properties.


### DEVELOPMENT OF ACID-SENSITIVE PLATINUM(II) COMPLEXES WITH PROTEIN-BINDING PROPERTIES

M. T. Schütte[1], R. Mülhaupt[1], and F. Kratz[2*]

[1] Institute of Macromolecular Chemistry, University of Freiburg, Stefan-Meier-Straße 31, 79104 Freiburg, Germany
[2] Tumor Biology Center, Department of Medical Oncology, Clinical Research, Breisacher Straße 117, 79106 Freiburg, Germany

### Abstract

Four new protein-binding platinum(II) complexes, **10**, **11**, **21**, **22**, in which the dichloroplatinum moiety is coordinated either to a carbon-substituted or a nitrogen-substituted ethylene diamino ligand, were prepared in ten-step syntheses. According to pH-dependent stability studies with strictly related compounds, **11** and **22** exhibit acid-sensitive properties.

### 1. Introduction

Cisplatin and carboplatin are potent antineoplastic drugs which are widely used in anticancer chemotherapy. They show their best results in the treatment of testicular and ovarian carcinoma and are also effective against bladder tumours and tumours of the head and neck [1]. However, both platinum complexes do not accumulate in tumour tissues but are distributed rapidly throughout the body after parenteral administration causing several side-effects such as nephrotoxicity, myelotoxicity, ototoxicity, nausea and vomiting [1].
Hence, a major goal in anticancer chemotherapy is the development of drug delivery systems that transport the drug to the tumour while largely sparing the healthy tissues of the body. In recent years, numerous preclinical studies have shown that macromolecules such as synthetic polymers and serum proteins are taken up by tumour tissue [2]. These polymers can accumulate in solid tumours due to an enhanced vascular permeability of tumour blood vessels for circulating macromolecules combined with a lacking lymphatic drainage system in the tumour.
Following this targeting strategy, we have developed several drug protein conjugates with organic anticancer drugs, e.g. with anthracyclines or the alkylating agent chlorambucil [3-10]. In order to bind an active *cis*-configurated platinum(II) complex to the surface of a macromolecular carrier, we set out to design appropriate ligands that are able to form a complex with platinum(II) and are also able to bind to the respective macromolecule as shown by the general structure in Figure 1:

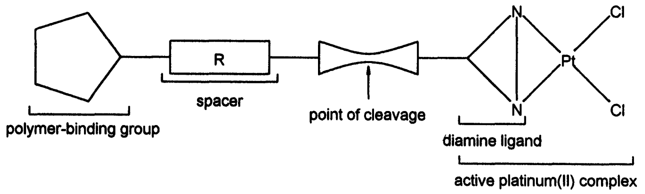

Figure 1: General structure of platinum(II) complexes capable of binding to macromolecules

In 1998, we reported on two platinum complexes in which the platinum moiety was bound to a spacer through a stable aromatic or aliphatic ester bond and also contained a maleimide group that is able to react specifically with thiolated macromolecules (see Figure 2) [11].

* Corresponding author: tel.: +49 761 206 2176; fax: +49 761 206 1899; e-mail· felix@tumorbio.uni-freiburg.de

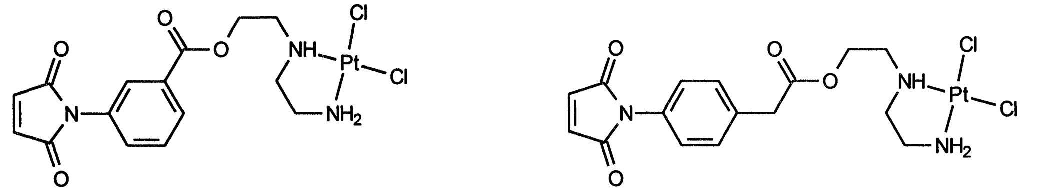

Figure 2: Structures of two platinum complexes with maleimide groups and ester bonds

However, recent studies in our group have revealed that the chemical bond between the drug and the carrier plays a crucial role for *in vitro* and *in vivo* activity. In our protein conjugates realised with anthracyclines or alkylating agents, an *acid-sensitive* linker was used which acts as a predetermined breaking-point releasing the protein-bound drug either in the acidic environment of tumour tissue or in the acidic endosomal or lysosomal compartments after cellular uptake of the conjugate by the tumour cell [3,10].

In subsequent biological studies, we were able to establish a correlation between the acid-sensitivity of the chemical link and the antitumour activity of the conjugates: Drug protein conjugates in which the drug was bound to the protein through an acid-sensitive carboxylic hydrazone bond showed high *in vitro* and *in vivo* activity, whereas conjugates containing an ester or amide bond showed only marginal efficacy [4-7,9].

Carboxylic hydrazones have proven very useful as predetermined breaking-points because they are sufficiently stable at pH 7.4 – thus preventing the drug from being released in the blood stream – and they also show acid-sensitive properties in a pH-range which is common in most tumour tissues and in relevant organelles of tumour cells (pH range 4-6.5).

For these reasons, we prepared four maleimide containing platinum complexes with two different ligand systems that incorporate a carboxylic hydrazone bond. The synthesis and characterisation of these complexes are described in the following.

### 2. Results and Discussion

### 2.1. Platinum complexes with a N-(2-hydroxyethyl)-ethylene diamine ligand

The method of preparing the platinum(II) complexes **10** and **11**, which contain the N-(2-hydroxyethyl)-ethylene diamine ligand as the complexing moiety, is depicted in scheme 1. The starting compound **1** was synthesised as described previously [11]. In a first step, **1** was reacted with 4-formylbenzoyl chloride (**2**) and 4-acetylbenzoyl chloride (**3**), respectively. The resulting esters **4** and **5** could then be directly transformed into the carboxylic hydrazones **6** and **7** by reaction with 3-maleimidobenzoylhydrazide trifluoroacetate which was prepared in five steps according to the method of Beyer et al. [12].

In the next step, the Boc-protecting groups (Boc = *tert.*-butoxycarbonyl) were removed. It proved best to use pure trifluoroacetic acid in the absence of water, thus preventing a cleavage of the acid-sensitive hydrazone bond. Isolation of **8** and **9** was achieved by adding diethyl ether resulting in the precipitation of the amine salts. In the last step, **8** and **9** were treated with potassium tetrachloroplatinate(II) in a mixture of DMF and water until the solutions became bright yellow. Upon addition of excess water the platinum complexes **10** and **11** precipitated as pale yellow solids which were collected by filtration and dried in vacuo.

### 2.2. Platinum complexes with a 2,3-diaminopropionic acid ligand

Two further protein-binding platinum complexes containing the 2,3-diaminopropionic acid ligand were synthesised as depicted in scheme 2. Initially, the starting material, D,L-2,3-diaminopropionic acid hydrochloride, was Boc-protected by reaction with di-*tert.*-butyldicarbonate in a methanol/KOH/water mixture. The resulting carboxylic acid **12** was then reacted with 4-hydroxybenzaldehyde (**13**) and 4-hydroxyacetophenone (**14**), respectively, to form the esters **15** and **16**.

The next reaction steps were performed in analogy to those described for the platinum complexes **10** and **11**. In the last step, the diamine trifluoroacetates were dissolved in DMF/water and neutralised with diluted KOH. Subsequently, potassium tetrachloroplatinate(II) was added, and the solutions were stirred at room temperature until the colour changed to yellow. Upon addition of water, **21** and **22** were obtained as pale yellow solids.

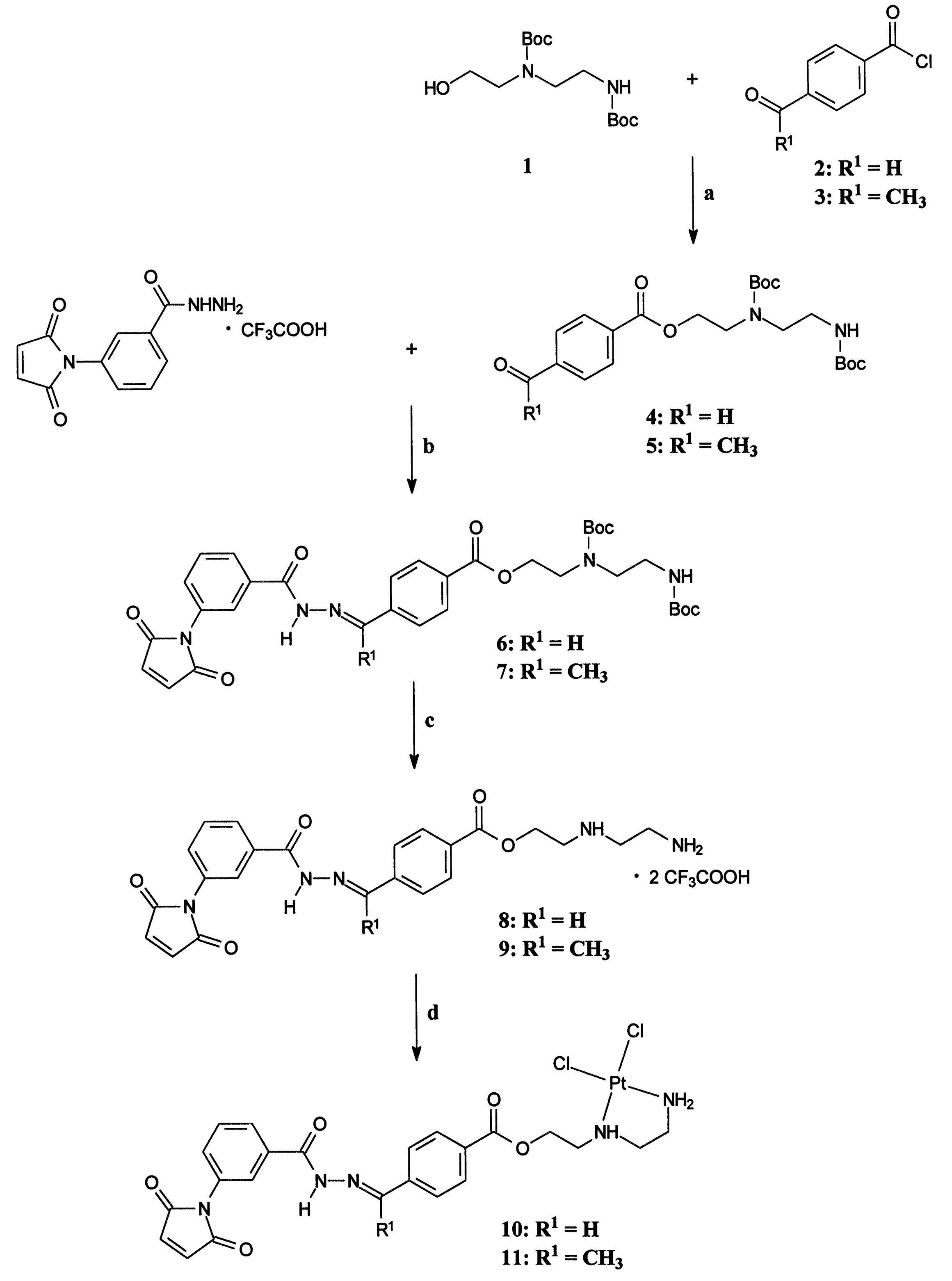

**a: $Et_3N$ / THF; b: THF, c: 1. $CF_3COOH$, 2. $Et_2O$, d: $K_2PtCl_4$, DMF / $H_2O$**

Scheme 1

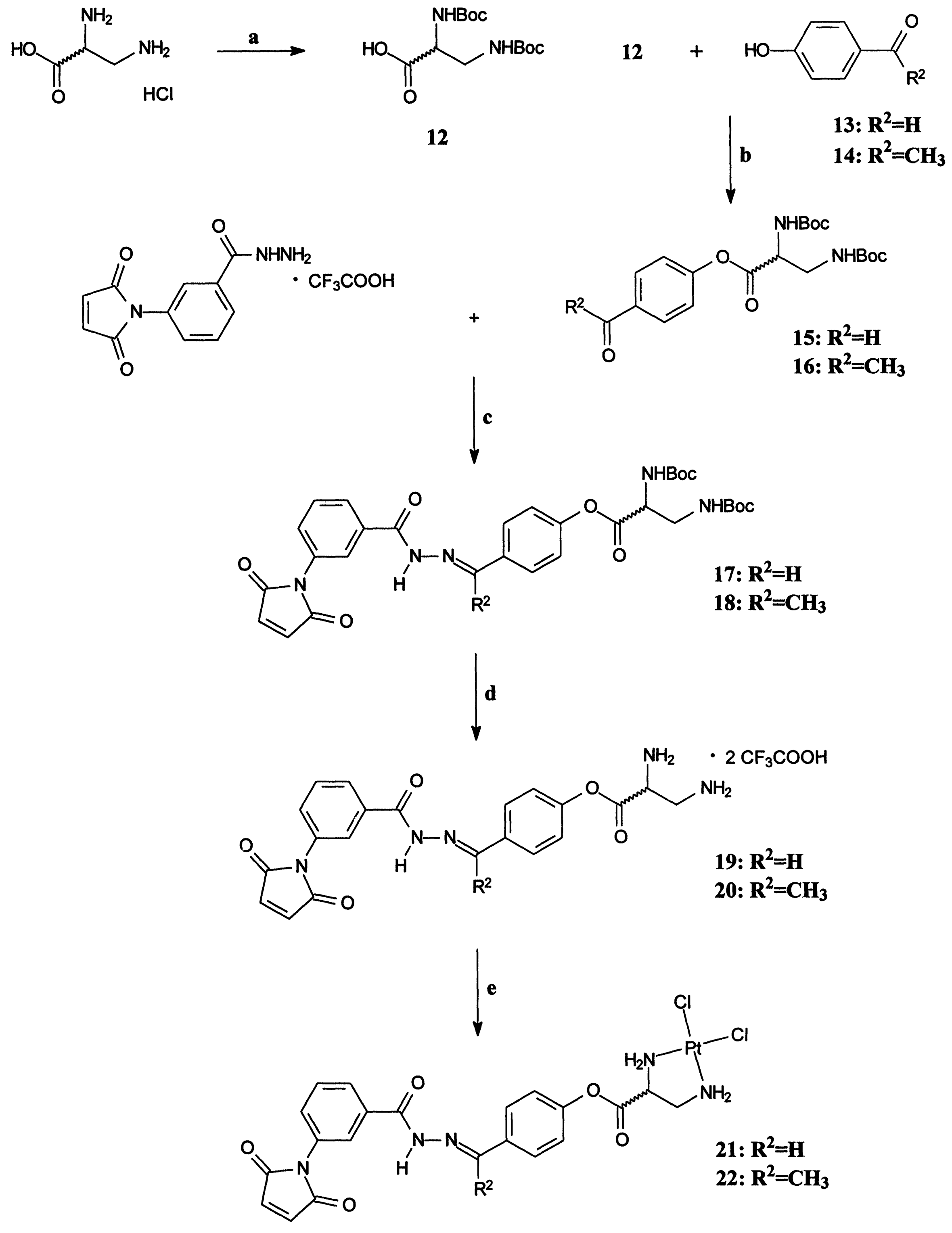

**a: $(Boc)_2O$, MeOH/KOH/$H_2O$; b: DCC, DMAP, THF; c: THF; d: 1. $CF_3COOH$, 2. $Et_2O$; e: $K_2PtCl_4$, DMF / $H_2O$**

Scheme 2

### 2.3. Characterisation

All synthesised compounds were characterised through $^1H$ and $^{13}C$ NMR spectroscopy and additionally by elemental analysis (see Experimental Section). Furthermore, mass spectra of the four platinum complexes were recorded.

NMR spectroscopy of the platinum complexes **10**, **11**, **21** and **22** revealed the characteristic singlet peaks of the introduced maleimide group at 7.2 ppm for the protons of the double bond, the singlets at 8.5-8.6 ppm for the aldehyde hydrazone protons of **10** and **21** as well as the singlets at 2.4 ppm for the methyl groups of **11** and **22**. Additionally, all four complexes showed the typical signals of the maleimide group in the $^{13}C$ NMR spectra at 134-135 ppm for the carbon atoms of the double bond and at 169-170 ppm for the carbonyl group.

In the $^1H$ NMR and $^{13}C$ NMR spectra of the platinum complexes as well as of the carboxylic hydrazone compounds (**6-9** and **17-20**) one set of signals was observed in DMSO-$d_6$ indicating the presence of only one stereoisomer. From a steric point of view, E-isomers are favoured, and we therefore tentatively propose that this stereoisomer is present which is in accordance with studies on simple hydrazones [13].

The ESI mass spectra of the four complexes reveal the characteristic molecular peaks ($M^+$+1) at 716, 730, 688 and 702 mass units for **10**, **11**, **21** and **22**. In addition, the peaks $M^+$+1-Cl and $M^+$+1-2Cl are distinctly visible which correspond to the successive loss of the coordinatively bound chlorides. As an example, the mass spectrum of **21** in the range of 600 to 700 mass units is shown in Figure 3.

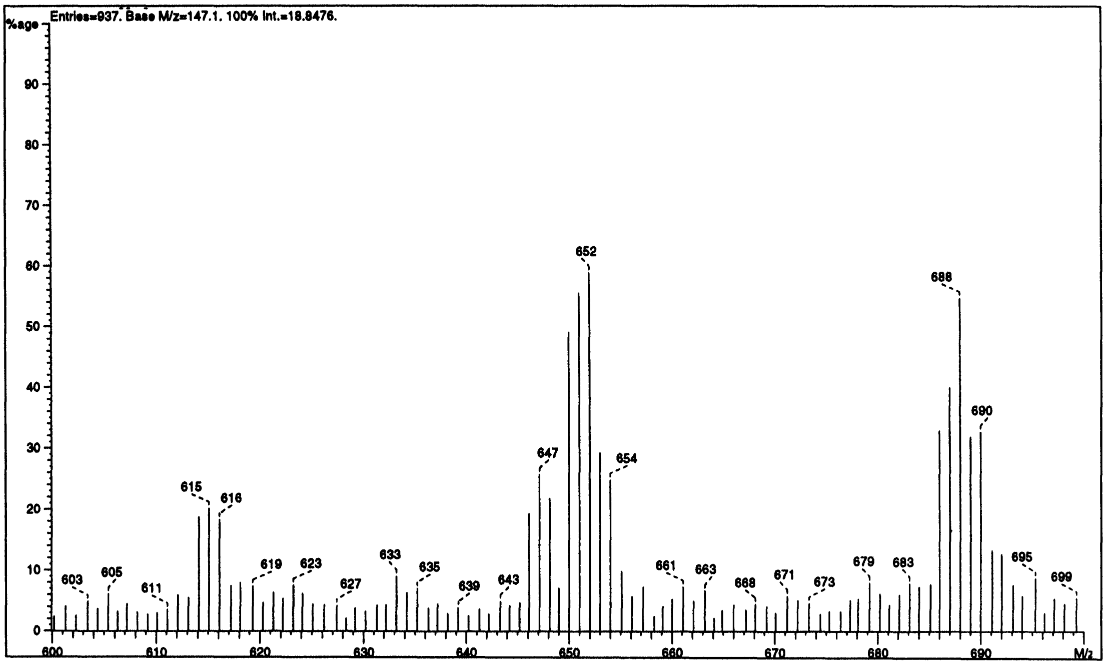

Figure 3: Mass spectrum of the platinum complex **21** in the range of 600-700 mass units

### 3. pH-dependent hydrolysis studies

As mentioned above, the acid-sensitivity of the link between the drug and the carrier is an important parameter for the antitumour activity of the conjugates. Hence, we wanted to perform pH-dependent hydrolysis studies to determine the acid-sensitivity of our synthesised complexes. Unfortunately, the platinum complexes **10**, **11**, **21** and **22** could not be used for this purpose due to their poor water-solubility. The precursors of the platinum complexes are not suited either because the maleimide group is not sufficiently stable in aqueous solutions over a period of several hours which is desired for performing hydrolysis studies.

Therefore, we synthesised the model compounds **27** and **28** which are strictly related to **17** and **18**, but contain a non-hydrolysable succinic imide group instead of the maleimide group. The synthesis of the model compounds is depicted in scheme 3.

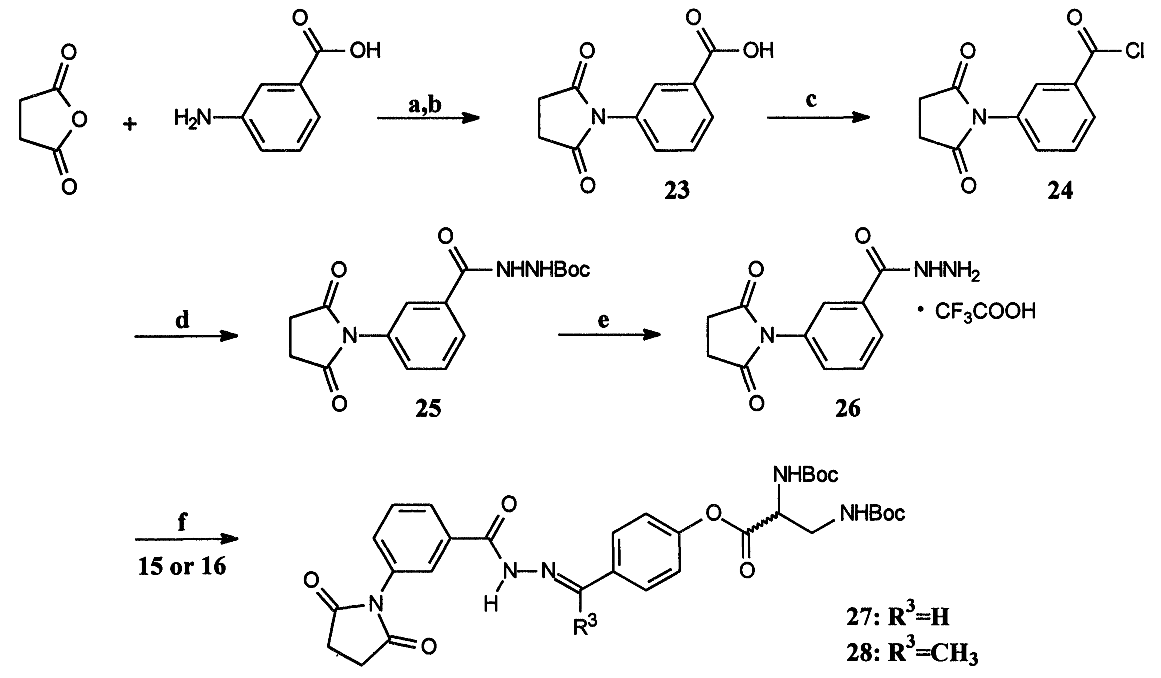

a: THF; b: $Ac_2O$, NaOAc; c: $SOCl_2$, toluene; d: $H_2NNHBoc$, THF; e: 1. $CF_3COOH$, 2.$Et_2O$; f: EtOAc

Scheme 3: Synthesis of the model compounds **27** and **28**

**27** and **28** were not deprotected by acid cleavage because the resulting amine salts were difficult to analyse by reverse phase HPLC using simple mixtures of water and an organic solvent. In contrast, **27** and **28** showed definite elution profiles on a C18 reverse phase HPLC-column (eluent: acetonitrile/water 1:1; retention time for **27** was 7.9 minutes and 8.9 minutes for **28**).

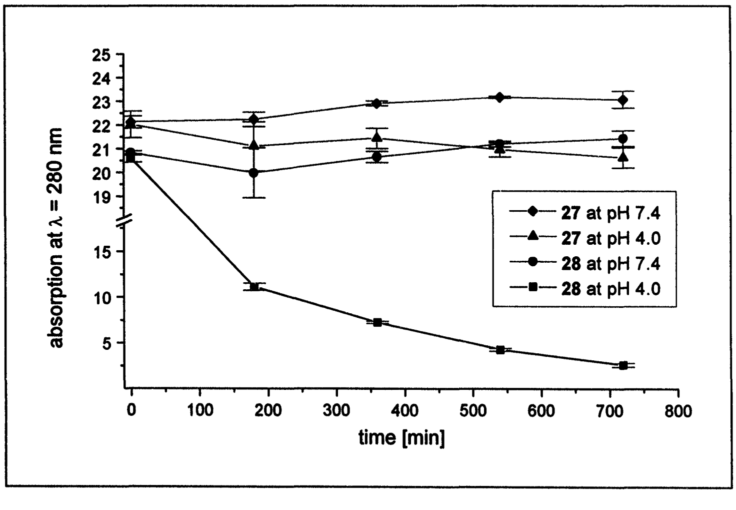

Figure 4: pH-dependent stability of the model compounds **27** and **28** at pH 7.4 and pH 4.0 over a range of 12 hours.

Hydrolysis studies were carried out as follows: Stock solutions of **27** and **28** ($c = 6 \cdot 10^{-5}$ mol/l) in acetonitrile were prepared. 520 µl of the respective stock solution were diluted with 780 µl of buffer A (0.004 M sodium phosphate, 0.15 M sodium chloride, pH 7.4) or buffer B (0.004 M sodium phosphate, 0.15 M sodium chloride, pH 4.0). Subsequently, 50 µl samples were analysed at t = 0, 3, 6, 9 and 12 h by HPLC (column: Lichrosorb RP-18 (5µm), l = 30 cm, from Merck KGaA, Darmstadt, FRG) at λ = 280 nm using an autosampler (determinations were carried out in triplicate). The rates of hydrolysis were measured by the decrease of the peak areas of **27** and **28** and are depicted in Figure 4.

Both **27** and **28** do not hydrolyse at pH 7.4. At pH 4.0, however, **28** hydrolyses rapidly with a half-life of approximately 3.5 hours. Interestingly, **27** does not exhibit pronounced acid-sensitive properties with less than 10% being hydrolysed at pH 4.0 after 12 hours.

In summary, we have prepared four new protein-binding platinum complexes in which the dichloroplatinum moiety is coordinated either to a carbon-substituted or a nitrogen-substituted ethylene diamino ligand.

According to our pH-dependent stability studies with the strictly related model compounds **27** and **28**, the complexes containing an acetophenone benzoylhydrazone bond (**11** and **22**) exhibit acid-sensitive properties in contrast to the two complexes **10** and **21** that contain a benzaldehyde benzoylhydrazone bond.

Preliminary experiments have shown that all four platinum complexes can be coupled to thiol-group bearing macromolecules such as human serum albumin or polyethylene glycol-SH. Respective polymer conjugates with **10**, **11**, **21** and **22** will be tested for antitumour activity in order to obtain structure-activity relationships for the new platinum conjugates.

### Acknowledgements:

We wish to thank the Deutsche Forschungsgemeinschaft and the Schering Research Foundation for their support.

### 4. Experimental Section

### 4.1. General

All chemicals were purchased from Aldrich, Fluka and Merck KGaA. Melting points: Büchi 530; $^{1}$H NMR and $^{13}$C NMR: Bruker ARX 300 (internal standard: TMS); electron spray MS: Finnigan-MAT 312; elemental analysis: Perkin-Elmer elemental analyser 240; silica gel chromatography on silica gel 60 (0.063-0.100 mm) from Merck KGaA; TLC: silica coated plates 60 $F_{254}$ from Merck KGaA; organic solvents: p.a. grade and a gift from BASF AG or purchased from Merck KGaA, FRG; $K_2PtCl_4$ was kindly provided by Asta Medica, FRG.

### 4.2. Synthesis

**4-formylbenzoyl chloride (2), 4-acetylbenzoyl chloride (3)**

0.1 mol 4-carboxybenzaldehyde or 4-carboxyacetophenone was suspended in 300 ml toluene and treated with 0.3 mol thionyl chloride (36 g) under reflux (according to the method of Slotta and Kethur [14]) until the suspension cleared. Excess thionyl chloride was then removed in vacuo, the remaining solution filtered and evaporated to dryness. The yellow residue was crystallised from diethyl ether to yield 15.1 g (89.6 mmol, 89.6%) **2** or 12.6 g (69.1 mmol, 69.1%) **3** as yellow needles.

**2**: $C_8H_5ClO_2$ (168.45 g/mol), m.p.: 54 °C, calc.: C: 56.99%, H: 2.97%, Cl: 21.04%, found: C: 56.71%, H: 3.22%, Cl: 20.97%, $^{1}$H NMR (300 MHz, $CDCl_3$): δ = 10.0 (s, 1H, CHO), 8.1 (d, 2H, arom. H, $^{3}J_{23}$ = $^{3}J_{65}$ = 12.05 Hz), 7.9 (d, 2H, arom. H, $^{3}J_{32}$ = $^{3}J_{56}$ = 12.05 Hz), $^{13}$C NMR (75.4 MHz, $CDCl_3$): δ = 189.9 (CHO), 163.4 (COCl), 135.9/132.6/127.0/126.6 (arom. C)

**3**: $C_9H_7ClO_2$ (182.45 g/mol), m.p.: 67 °C, calc.: C: 59.19%, H: 3.84%, Cl: 19.43%, found: C: 59.06%, H: 3.76%, Cl: 19.15%, $^{1}$H NMR (300 MHz, $CDCl_3$): δ = 8.2 (d, 2H, arom. H, $^{3}J_{23}$ = $^{3}J_{65}$ = 8.66 Hz), 8.1 (d, 2H, arom. H, $^{3}J_{32}$ = $^{3}J_{56}$ = 8.66 Hz), 2.7 (s, 3H, $CH_3$), $^{13}$C NMR (75.4 MHz, $CDCl_3$): δ = 197.0 ($CH_3CO$), 167.9 (COCl), 141.8/136.5/131.5/128.6 (arom. C), 27.0 ($CH_3$)

**4-[3,6-bis-(*tert.*-butoxycarbonyl)-3,6-diazahexyloxycarbonyl]-benzaldehyde (4), 4-[3,6-bis-(*tert.*-butoxycarbonyl)-3,6-diazahexyloxycarbonyl]-acetophenone (5)**

16.45 mmol **1** and 16.45 mmol of either **2** or **3** were dissolved in 30 ml THF and 16.45 mmol pyridine (1.3 g) added dropwise under vigorous stirring at room temperature. After 12 h pyridine hydrochloride was filtered off and the filtrate evaporated to dryness in vacuo. The crude product was dissolved in a minimal amount of ethyl acetate and purified by column chromatography (silica gel, ethyl acetate / n-hexane 1:3) to yield 6.82 g (15.64 mmol, 95.1%) of **4** or 6.79 g (15.09 mmol, 91.7%) of **5**.

**4**: $R_f$ = 0.44 (ethyl acetate / n-hexane 1:2), $C_{22}H_{32}N_2O_7$ (436 g/mol), m.p.: 84 °C, calc.: C: 60.55%, H: 7.34%, N: 6.42%, found: C: 60.79%, H: 7.24%, N: 6.46%, $^{1}$H NMR (300 MHz, $CDCl_3$): δ = 10.1 (s, 1H, CHO), 8.2 (d, 2H, $H_{ar}$-3/$H_{ar}$-5, $^{3}J_{32}$ = $^{3}J_{56}$ = 8.32 Hz), 7.9 (d, 2H, $H_{ar}$-2/$H_{ar}$-6, $^{3}J_{23}$ = $^{3}J_{65}$ = 8.32 Hz), 4.9 (d, 1H, NH), 4.5 (s, 2H, $OCH_2$), 3.6/3.4/3.3 (3s, 6H, 3 $NCH_2$), 1.4 (s, 18H, 6 $CH_3$), $^{13}$C NMR (75.4 MHz, $CDCl_3$): δ = 191.6 (CHO), 165.4 (Ar-COO), 155.5 (NCOO), 139.3/134.9/130.3/129.5 (arom. C), 80.5 ($C(CH_3)_3$), 63.5 ($OCH_2$), 47.4/46.6/39.4 (3 $NCH_2$), 28.3 (6 $CH_3$)

**5**: $R_f$ = 0.43 (ethyl acetate / n-hexane 1:2), $C_{23}H_{34}N_2O_7$ (450 g/mol), m.p.: 84 °C, calc.: C: 61.33%, H: 7.56%, N: 6.22%, found: C: 61.08%, H: 7.31%, N: 6.08%, $^{1}$H NMR (300 MHz, $CDCl_3$):

$\delta$ = 8.1 (dd, 2H, $H_{ar}$-3/$H_{ar}$-5, $^3J_{32}$ = $^3J_{56}$ = 6.79 Hz, $^4J_{35}$ = 1.89 Hz), 7.9 (dd, 2H, $H_{ar}$-2/$H_{ar}$-6, $^3J_{23}$ = $^3J_{65}$ = 6.79 Hz, $^4J_{26}$ = 0.56 Hz), 4.8 (d, 1H, NH), 4.4 (s, 2H, $OCH_2$), 3.7/3.4/3.3 (3s, 6H, 3 $NCH_2$), 2.6 (s, 3H, $COCH_3$) 1.3 (s, 18H, 6 $CH_3$), $^{13}$C NMR (75.4 MHz, $CDCl_3$): $\delta$ = 197.5 ($COCH_3$), 165.5 (Ar-COO), 155.5 (NCOO), 140.3/133.7/130.0/128.2 (arom. C), 80.4 ($C(CH_3)_3$), 63.4 ($OCH_2$), 47.5/46.6/39.4 (3 $NCH_2$), 28.4 (6 $CH_3$), 26.9 ($COCH_3$)

**4-[3,6-bis-(*tert.*-butoxycarbonyl)-3,6-diazahexyloxycarbonyl]-benzaldehyde 3-maleimidobenzoylhydrazone (6), 4-[3,6-bis-(*tert.*-butoxycarbonyl)-3,6-diazahexyloxycarbonyl]-acetophenone 3-maleimidobenzoylhydrazone (7)**

4.59 mmol 3-maleimidobenzoylhydrazide trifluoroacetate and 4.59 mmol of **4** or **5** were suspended in 40 ml ethyl acetate and heated at 50 °C until the suspension cleared. After stirring for 2 h at room temperature, the solvent was removed in vacuo and the crude product purified by column chromatography (silica gel, ethyl acetate / n-hexane 1:1) to yield 1.5 g (2.31 mmol, 50.3%) **6** or 1.3 g (1.96 mmol, 42.7%) **7**.

**6:** $R_f$ = 0.24 (ethyl acetate / n-hexane 2:1), $C_{33}H_{39}N_5O_9$ (649 g/mol), m.p.: 84 °C, calc.: C: 61.02%, H: 6.01%, N: 10.79%, found: C: 60.62%, H: 6.20%, N: 10.53%, $^1$H NMR (300 MHz, DMSO-$d_6$): $\delta$ = 12.1 (s, 1H, C=NNH), 8.5 (s, 1H, N=CH), 8.1-7.9 (m, 4H, arom. H), 7.7 (m, 2H, arom. H), 7.6 (m, 2H, arom. H), 7.2 (s, 2H, HC=CH), 6.8 (bs, 1H, $CH_2NH$), 4.4 (m, 2H, $OCH_2$), 3.5/3.3/3.1 (3s, 6H, 3 $NCH_2$), 1.3 (s, 18H, 6 $CH_3$), $^{13}$C NMR (75.4 MHz, DMSO-$d_6$): $\delta$ = 170.1 (OC-CH=CH-CO), 165.5 (Ar-COO), 162.8 (C=NNH-CO), 155.9 (C=N) 155.5 (NCOO), 147.1/139.0/135.2/132.3/130.9/130.1/129.5/127.5/127.1/126.6 (arom. C), 134.3 (HC=CH) 79.2/78.0 (2 $C(CH_3)_3$), 63.4 ($OCH_2$), 47.0/45.8/38.5 (3 $NCH_2$), 28.4 (6 $CH_3$)

**7:** $R_f$ = 0.38 (ethyl acetate / n-hexane 2:1), $C_{34}H_{41}N_5O_9$ (663 g/mol), m.p.: 82 °C, calc.: C: 61.54%, H: 6.18%, N: 10.56%, found: C: 60.78%, H: 6.08%, N: 10.04%, $^1$H NMR (300 MHz, DMSO-$d_6$): $\delta$ = 10.9 (s, 1H, C=NNH), 8.1-7.5 (m, 8H, arom. H), 7.2 (s, 2H, HC=CH), 6.8 (s, 1H, $CH_2NH$), 4.4 (s, 2H, $OCH_2$), 3.6/3.4/3.0 (3m, 6H, 3 $NCH_2$), 2.4 (N=C-$CH_3$), 1.4 (s, 9H, 3 $CH_3$), 1.3 (s, 9H, 3 $CH_3$), $^{13}$C NMR (75.4 MHz, DMSO-$d_6$): $\delta$ = 169.8 (OC-CH=CH-CO), 165.2 (Ar-COO), 163.2 (C=NNH-CO), 155.6 (C=N), 154.6 (NCOO), 142.3/131.6/130.0/129.2/128.8/126.5 (arom. C), 134.7 (HC=CH) 78.8 ($C(CH_3)_3$), 59.7 ($OCH_2$), 46.7/45.5/38.1 (3 $NCH_2$), 28.4 (6 $CH_3$), 28.0 (N=C-$CH_3$)

**4-(3,6-diazahexyloxycarbonyl)-benzaldehyde 3-maleimidobenzoylhydrazone bis-trifluoroacetate (8), 4-(3,6-diazahexyloxycarbonyl)-acetophenone 3-maleimidobenzoylhydrazone bis-trifluoroacetate (9)**

1.54 mmol of **6** or **7** were treated with 2 ml trifluoroacetic acid for a few minutes at room temperature until a clear viscous yellow solution was formed. The solution was triturated with 25 ml diethyl ether to produce **8** or **9** as precipitates, which were separated by filtration and washed twice with 25 ml diethyl ether. The products were dried in vacuo to yield 0.93 g (1.37 mmol, 89.2%) of **8** or 0.84 g (1.22 mmol, 78.9%) of **9** as pale yellow powders.

**8:** $C_{27}H_{25}N_5O_9F_6$ (677 g/mol), m.p.: 92 °C, calc.: C: 47.86%, H: 3.69%, N: 10.34%, found: C: 46.25%, H: 3.71%, N: 9.72%, $^1$H NMR (300 MHz, DMSO-$d_6$): $\delta$ = 12.2 (s, 1H, C=NNH), 9.3 (bs, 3H, $NH_3^+$), 8.5 (s, 1H, N=CH), 8.2-7.7 (m, 8H, arom. H), 7.6 (m, 2H, $NH_2^+$), 7.2 (s, 2H, HC=CH), 4.5 (s, 2H, $OCH_2$), 3.6/3.4/3.2 (3m, 6H, 3 $NCH_2$), $^{13}$C NMR (75 4 MHz, DMSO-$d_6$): $\delta$ = 169.7 (OC-CH=CH-CO), 165.1 (Ar-COO), 162.4 (C=NNH-CO), 158.8 (q, $CF_3COOH$, J = 30.2 Hz), 156.1 (C=N), 147.7/146.8/138.9/133.9/131.9/130.1/129.1/127.0/126.7/126.2 (arom. C), 134.7 (HC=CH), 116.7 (q, $CF_3COOH$, J = 294.1 Hz), 64.2 ($OCH_2$), 45.8/44.2/35.1 (3 $NCH_2$)

**9:** $C_{28}H_{27}N_5O_9F_6$ (691 g/mol), m.p.: 125 °C, calc.: C: 48.63%, H: 3.91%, N: 10.13%, found: C: 45.98%, H: 3.82%, N: 9.71%, $^1$H NMR (300 MHz, DMSO-$d_6$): $\delta$ = 10.9 (s, 1H, C=N-NH), 9.4 (bs, 3H, $NH_3^+$), 8.4-7.8 (m, 8H, arom. H), 7.6 (m, 2H, $NH_2^+$), 7.2 (s, 2H, HC=CH), 4.6 (s, 2H, $OCH_2$), 3.5/3.3/3.2 (3m, 6H, 3 $NCH_2$), 2.4 (N=C-$CH_3$), $^{13}$C NMR (75.4 MHz, DMSO-$d_6$): $\delta$ =169.8 (OC-CH=CH-CO), 165.2 (Ar-COO), 163.4 (C=NNH-CO), 158.9 (q, $CF_3COOH$, J = 33.9 Hz), 155.6 (C=N) 142.7/140.3/132.8/131.7/129.9/129.7/128.8/127.2/126.5 (arom. C), 134.7 (HC=CH), 116.4 (q, $CF_3COOH$, J = 295.0 Hz), 60.6 ($OCH_2$), 45.9/44.2/35.1 (3 $NCH_2$), 27.2 (N=C-$CH_3$)

**N-[4-[3-(3-maleimidobenzoyl)-2,3-diazaprop-1-en-1-yl]-benzoyloxyethyl]-ethylenediaminodichloroplatin(II) (10), N-[4-[4-(3-maleimidobenzoyl)-3,4-diazabut-2-en-2-yl]-benzoyloxyethyl]-ethylenediaminodichloroplatin(II) (11)**

0.14 mmol $K_2PtCl_4$ were dissolved in 20 ml of DMF/water (80:20) and treated under stirring in the dark with 0.14 mmol of **8** or **9** dissolved in 10 ml DMF/water. The solution was stirred at room temperature until the colour changed from red to yellow. Upon addition of 40 ml of water the platinum complexes **10** and **11** precipitated. The suspension was kept cool for 5 h to 4 °C to complete precipitation. The solids were filtered off and washed twice with water, then twice with THF, twice with diethyl ether and finally dried in vacuo to yield 71.5 mg (0.1 mmol, 71.4%) **10** or 80.2 mg (0.11 mmol, 78.6%) **11**.

**10:** $C_{23}H_{23}N_5O_5PtCl_2$ (715 g/mol), m.p.: decomp. > 250 °C, calc.: C: 38.60%, H: 3.22%, N: 9.79%, Pt: 27.29%, Cl: 9.92%, found: C: 37.28%, H: 3.58%, N: 9.13%, Pt: 26.79%, Cl: 9.05%, $^1$H NMR (300 MHz, DMSO-$d_6$): $\delta$ = 12.1 (s, 1H, C=NNH), 8.6 (s, 1H, N=CH), 8.2-7.5 (m, 8H, arom. H), 7.2 (s, 2H, HC=CH), 4.6 (m, 2H, $OCH_2$), $^{13}$C NMR (75.4 MHz, DMSO-$d_6$): $\delta$ = 169.7 (OC-CH=CH-CO), 165.0 (Ar-COO), 162.3 (C=NNH-CO), 156.1 (C=N), 134.7 (HC=CH), 61.5 ($OCH_2$), 54.3/49.6/35.7 (3 $NCH_2$), ESI-MS (3.4 kV, DMF/MeOH 7:1, rel. intensity): *m/z* 716 ($M^+$+1, 14), 680 ($M^+$-Cl, 12), 643 ($M^+$-2Cl, 8)

**11:** $C_{24}H_{25}N_5O_5PtCl_2$ (729 g/mol), m.p.: decomp. > 250 °C, calc.: C: 39.51%, H: 3.43%, N: 9.60%, Pt: 26.76%, Cl: 9.73%, found: C: 38.24%, H: 3.67 %, N: 8.92%, Pt: 25.89%, Cl: 9.11%, $^1$H NMR (300 MHz, DMSO-$d_6$): δ = 10.9 (s, 1H, C=N-NH), 8.4-7.8 (m, 8H, arom. H), 7.2 (s, 2H, HC=CH), 2.4 (s, 3H, N=C-CH$_3$), $^{13}$C NMR (75.4 MHz, DMSO-$d_6$): δ = 169.8 (OC-CH=CH-CO), 165.2 (Ar-COO), 162.5 (C=NNH-CO), 155.7 (C=N), 134.7 (HC=CH), 62.1 (OCH$_2$), 56.8/50.2/37.1 (3 NCH$_2$), ESI-MS (3.4 kV, DMF/MeOH 7:1, rel. intensity): *m/z* 730 ($M^+$+1, 18), 694 ($M^+$-Cl, 48), 657 ($M^+$-2Cl, 45)

**N,N'-bis-(*tert.*-butoxycarbonyl)-D,L-2,3-diaminopropionic acid (12)**

0.14 mol di-*tert.*-butyldicarbonate (30.96 g) were added to a solution of 71.14 mmol D,L-2,3-diaminopropionic acid hydrochloride (10 g) in a mixture of 100 ml methanol and 50 ml 4N KOH and the reaction mixture was stirred for 12 h at room temperature. After removing methanol in vacuo, the aqueous solution was acidified to pH 1 with concentrated hydrochloric acid and extracted with ethyl acetate. The organic layer was dried over sodium sulfate and concentrated in vacuo to yield 19 g (62.5 mmol, 87.9%) of **12** as a white solid. $C_{13}H_{24}N_2O_6$ (304 g/mol), m.p.: 145 °C, calc.: C: 51.32%, H: 7.89%, N: 9.21%, found: C: 50.96%, H: 8.16%, N: 9.58%, $^1$H NMR (300 MHz, DMSO-$d_6$): δ = 12.3 (bs, 1H, COOH), 6.8 (d, 1H, CHNH), 6.7 (t, 1H, CH$_2$NH) 3.9 (q, 1H, CH), 3.2 (t, 2H, CH$_2$), 1.4/1.3 (2s, 18H, 6 CH$_3$), $^{13}$C NMR (75.4 MHz, DMSO-$d_6$): δ = 173.1 (COOH), 156.5/156.1 (2 NCOO), 79.0/78.8 (2 C(CH$_3$)$_3$), 54.6 (CH), 42.0 (CH$_2$), 29.0 (6 CH$_3$)

**4-[N,N'-bis-(*tert.*-butoxycarbonyl)-D,L-2,3-diaminopropanoyloxy]-benzaldehyde (15), 4-[N,N'-bis-(*tert.*-butoxycarbonyl)-D,L-2,3-diaminopropanoyloxy]-acetophenone (16)**

9.87 mmol **12**, 9.0 mmol of **13** or **14** and 0.1 mmol N,N-dimethylaminopyridine (12 mg) were dissolved in 100 ml THF and 10 mmol N,N'-dicyclohexylcarbodiimide (2.1 g), dissolved in 20 ml THF, were then added dropwise under stirring at 4 °C. The solution was stirred for 2 h at 4 °C, then for further 12 h at room temperature and finally dicyclohexyl urea was filtered off. The filtrate was evaporated to dryness, dissolved in 100 ml ethyl acetate and washed twice with 100 ml of a saturated NaHCO$_3$ solution and then twice with 100 ml water. The organic layer was dried over sodium sulfate and concentrated to a volume of 50 ml. n-hexane was added until a slight turbidity remained. In the cold at 4 °C, colourless crystals formed yielding 2.97 g (7.28 mmol, 73.8%) of **15** or 1.80 g (4.26 mmol, 43.2%) of **16**.

**15:** $C_{20}H_{28}N_2O_7$ (408 g/mol), m.p.: 98 °C, calc.: C: 58.82%, H: 6.86%, N: 6.86%, found: C: 58.59%, H: 7.15%, N: 6.87%, $^1$H NMR (300 MHz, DMSO-$d_6$): δ = 10.0 (s, 1H, CHO), 8.0 (d, 2H, arom. H, J = 8.67 Hz), 7.3 (m, 3H, 2 arom. H/CHNH), 7.0 (t, 1H, CH$_2$NH), 4.3 (q, 1H, CH), 3.4 (m, 2H, CH$_2$), 1.4/1.3 (2s, 18H, 6 CH$_3$), $^{13}$C NMR (75.4 MHz, DMSO-$d_6$): δ = 192.8 (CHO), 170.3 (CHCO), 156.5/156.2 (2 NCOO), 155.9/134.9/131.9/123.4 (arom. C), 79.7/79.1 (2 C(CH$_3$)$_3$), 55.2 (CH), 41.6 (CH$_2$), 29.0 (6 CH$_3$)

**16:** $C_{21}H_{30}N_2O_7$ (422 g/mol), m.p.: 110 °C, calc.: C: 59.72%, H: 7.11%, N: 6.64%, found: C: 60.40%, H: 7.22%, N: 6.79%, $^1$H NMR (300 MHz, DMSO-$d_6$): δ = 8.0 (d, 2H, $H_{ar}$-2/$H_{ar}$-6, $^3J_{23}$ = $^3J_{65}$ = 8.67 Hz), 7.4 (d, 1H, CHNH), 7.3 (d, 2H, $H_{ar}$-3/$H_{ar}$-5, $^3J_{32}$ = $^3J_{56}$ = 8.67 Hz), 7.0 (m, 1H, CH$_2$NH), 4.3 (m, 1H, CH), 3.4 (m, 2H, CH$_2$), 2.6 (s, 3H, COCH$_3$), 1.4/1.3 (2s, 18H, 6 CH$_3$), $^{13}$C NMR (75.4 MHz, DMSO-$d_6$): δ = 197.1 (COCH$_3$), 169.7 (CHCO), 155.8/155.6 (2 NCOO), 154.3/134.8/130.1/122.1 (arom. C), 79.0/78.5 (2 C(CH$_3$)$_3$), 54.5 (CH), 41.0 (CH$_2$), 28.3/28.2 (6 CH$_3$), 26.9 (COCH$_3$)

**4-[N,N'-bis-(*tert.*-butoxycarbonyl)-D,L-2,3-diaminopropanoyloxy]-benzaldehyde 3-maleimido-benzoylhydrazone (17)**

3.68 mmol 3-maleimidobenzoylhydrazide trifluoroacetate and 3.68 mmol **15** were suspended in 30 ml ethyl acetate and heated at 50 °C until the suspension cleared. The product precipitated in the heat, was filtered off and crystallised from methanol to yield 1.88 g (3.03 mmol, 82.3%) **17**.

$C_{31}H_{35}N_5O_9$ (621 g/mol), m.p.: 224 °C, calc.: C: 59.90%, H: 5.64%, N: 11.27%, found: C: 60.58%, H: 5.34%, N: 11.51%, $^1$H NMR (300 MHz, DMSO-$d_6$): δ =12.0 (s, 1H, C=NNH), 8.5 (s, 1H, N=CH), 8.0-7.4 (m, 8H, arom. H), 7.3 (d, 1H, CHNH), 7.2 (s, 2H, HC=CH), 7.0 (t, 1H, CH$_2$NH), 4.3 (q, 1H, CH), 3.5 (m, 2H, CH$_2$), 1.4/1.3 (2s, 18H, 6 CH$_3$), $^{13}$C NMR (75.4 MHz, DMSO-$d_6$): δ = 169.7 (OC-CH=CH-CO), 169.6 (CHCO), 162.2 (C=NNH-CO), 155.5/155.3 (2 NCOO), 151.6 (C=N) 147.1/134.1/131.9/131.8/130.0/129.0/128.2/126.6/126.1/122.1 (arom. C), 134.7 (HC=CH), 78.7/78.2 (2 C(CH$_3$)$_3$), 54.2 (CH), 40.7 (CH$_2$), 28.1/28.0 (6 CH$_3$)

**4-[N,N'-bis-(*tert.*-butoxycarbonyl)-D,L-2,3-diaminopropanoyloxy]-acetophenone 3-maleimido-benzoylhydrazone (18)**

5.92 mmol 3-maleimidobenzoylhydrazide trifluoroacetate and 5.92 mmol **16** were suspended in 30 ml ethyl acetate and heated at 50 °C until the suspension cleared. The mixture was then concentrated to a minimal volume and separated by column chromatography (silica gel, ethyl acetate / n-hexane 3:2) to yield 2.22 g (3.50 mmol, 59.1%) **18** as a colourless powder.

$R_f$ = 0.35 (ethyl acetate / n-hexane 2:1), $C_{32}H_{37}N_5O_9$ (635 g/mol), m.p.: 93 °C, calc.: C: 60.47%, H: 5.83%, N: 11.02%, found: C: 60.72%, H: 5.45%, N: 11.17%, $^1$H NMR (300 MHz, DMSO-$d_6$): δ = 10.9 (s, 1H, C=NNH), 8.0-7.5 (m, 6H, arom. H), 7.4 (d, 1H, CHNH), 7.2 (m, 4H, arom. H/HC=CH), 7.1 (t, 1H, CH$_2$NH), 4.3 (m, 1H, CH), 3.4 (m, 2H, CH$_2$), 2.4 (s, 3H, N=C-CH$_3$), 1.4 (s, 9H, 3 CH$_3$), 1.3 (s, 9H, 3 CH$_3$), $^{13}$C NMR (75.4 MHz, DMSO-$d_6$): δ = 170.3 (OC-CH=CH-CO), 169.7 (CHCO), 163.1 (C=NNH-CO), 155.6/155.3 (NCOO), 151.4 (C=N), 135.7/131.7/129.7/128.9/127.6/127.0/126.3/121.5 (arom. C), 134.7 (HC=CH), 78.4/78.2 (C(CH$_3$)$_3$), 54.2 (CH), 40.8 (CH$_2$), 28.1 (6 CH$_3$), 28.0 (N=C-CH$_3$)

**4-(D,L-2,3-diaminopropanoyloxy)-benzaldehyde 3-maleimidobenzoylhydrazone bis-trifluoroacetate (19), 4-(D,L-2,3-diaminopropanoyloxy)-acetophenone 3-maleimidobenzoylhydrazone bis-trifluoroacetate (20)**

1.61 mmol **17** or **18** were treated at room temperature with 2 ml trifluoroacetic acid for a few minutes until a clear viscous yellow solution was formed. Under vigorous stirring, 25 ml diethyl ether were added resulting in the precipitation of **19** or **20**, which were separated by filtration and washed twice with 25 ml diethyl ether. The products were dried in vacuo to yield 0.88 g (1.36 mmol, 84.5%) **19** or 0.84 g (1.27 mmol, 78.7%) **20**.

**19:** $C_{25}H_{21}N_5O_9F_6$ (649 g/mol), m.p.: 66 °C, calc.: C: 46.22%, H: 3.24%, N: 10.79%, found: C: 44.62%, H: 3.21%, N: 9.79%, $^1$H NMR (300 MHz, DMSO-$d_6$): δ = 12.0 (s, 1H C=NNH), 8.7 (bs, 6H, 2 $NH_3^+$), 8.5 (s, 1H, N=CH), 8.0-7.3 (m, 8H, arom. H), 7.2 (s, 2H, HC=CH), 4.6 (t, 1H, CH), 3.5 (m, 2H, $CH_2$), $^{13}$C NMR (75.4 MHz, DMSO-$d_6$): δ = 170.7 (OC-CH=CH-CO), 166.5 (CHCO), 163.2 (C=NNH-CO), 159.5 (q, $CF_3$COOH, J = 32.7 Hz), 151.7 (C=N), 134.9 (HC=CH), 147.9-122.9 (arom. C), 117.7 (q, $CF_3$COOH, J = 297.2 Hz), 51.2 (CH), 39.1 ($CH_2$)

**20:** $C_{26}H_{23}N_5O_9F_6$ (663 g/mol), m.p.: 82 °C, calc.: C: 47.06%, H: 3.47%, N: 10.56%, found: C: 45.89%, H: 3.39%, N: 9.88%, $^1$H NMR (300 MHz, DMSO-$d_6$): δ = 11.0 (s, 1H, C=NNH), 9.0 (bs, 6H, 2 $NH_3^+$), 8.0-7.3 (m, 8H, arom. H), 7.2 (s, 2H, HC=CH), 4.7 (m, 1H, CH), 3.6 (m, 2H, $CH_2$), 2.4 (s, 3H, N=C-$CH_3$), $^{13}$C NMR (75.4 MHz, DMSO-$d_6$): δ = 169.8 (OC-CH=CH-CO), 165.6 (CHCO), 163.4 (C=NNH-CO), 159.5 (q, $CF_3$COOH, J = 33.1 Hz), 150.7 (C=N), 134.8 (HC=CH), 136.5-121.5 (arom. C), 117.7 (q, $CF_3$COOH, J = 296.9 Hz), 50.4 (CH), 40.2 ($CH_2$), 26.7 (N=C-$CH_3$)

**(R,S)-1-[4-[3-(3-maleimidobenzoyl)-2,3-diazaprop-1-en-1-yl]-phenoxycarbonyl]-ethylenediamino-dichloroplatin(II) (21), (R,S)-1-[4-[4-(3-maleimidobenzoyl)-3,4-diazabut-2-en-2-yl]-phenoxycarbonyl]-ethylenediaminodichloroplatin(II) (22)**

0.15 mmol **19** or **20** were dissolved in 10 ml DMF/water (80:20) and neutralised with 1.5 ml 0.1N KOH. These solutions were added to 0.15 mmol $K_2PtCl_4$, dissolved in 20 ml DMF/water (80:20), under stirring in the dark. Stirring was continued until the colour changed from red to yellow. Upon addition of 40 ml of water the platinum complexes **21** and **22** precipitated. The suspension was cooled for 5h to 4 °C to complete the precipitation. The precipitate was filtered off and washed twice with water, then twice with THF, twice with diethyl ether and finally dried in vacuo to yield 88.8 mg (0.13 mmol, 86.7%) **21** or 84.1 mg (0.12 mmol, 80.0%) **22**.

**21:** $C_{21}H_{19}N_5O_5PtCl_2$ (687 g/mol), m.p.: decomp. > 250 °C, calc.: C: 36.68%, H: 2.76%, N: 10.19%, Pt: 28.40%, Cl: 10.32%, found: C: 35.06%, H: 2.84%, N: 9.77%, Pt: 27.12%, Cl: 9.43%, $^1$H NMR (300 MHz, DMSO-$d_6$): δ = 12.1 (s, 1H, C=N-NH), 8.5 (s, 1H, N=CH), 8.0-7.5 (m, 8H, arom. H), 7.3 (d, 2H, $CHNH_2$), 7.2 (s, 2H, HC=CH), 6.8 (m, 2H, $CH_2NH_2$), 4.1 (m, 1H, CH), 3.1 (m, 2H, $CH_2$), $^{13}$C NMR (75.4 MHz, DMSO-$d_6$): δ = 169.7 (OC-CH=CH-CO), 166.1 (CHCO), 162.2 (C=NNH-CO), 148.4 (C=N), 134.7 (HC=CH), 60.3 (CH), 48.5 ($CH_2$), ESI-MS (3.8 kV, DMF/MeOH 7:1, rel. intensity): *m/z* 688 ($M^+$+1, 54), 652 ($M^+$-Cl, 59), 615 ($M^+$-2Cl, 20)

**22:** $C_{22}H_{21}N_5O_5PtCl_2$ (701 g/mol), m.p.: decomp. > 250 °C, calc.: C: 37.66%, H: 3.00%, N: 9.99%, Pt: 27.83%, Cl: 10.11%, found: C: 37.06%, H: 3.57%, N: 10.53%, Pt: 26.88%, Cl: 9.42%, $^1$H NMR (300 MHz, DMSO-$d_6$): δ = 10.9 (s, 1H, C=NNH), 8.1-7.5 (m, 8H, arom. H), 7.2 (s, 2H, HC=CH), 2.4 (s, 3H, N=C-$CH_3$), $^{13}$C NMR (75.4 MHz, DMSO-$d_6$): δ = 169.7 (OC-CH=CH-CO), 166.7 (CHCO), 163.2 (C=NNH-CO), 151.8 (C=N), 134.7 (HC=CH), 60.3 (CH), 48.5 ($CH_2$), 26.7 (N=C-$CH_3$), ESI-MS (3.4 kV, DMF/MeOH 7:1, rel. intensity): *m/z* 702 ($M^+$+1, 45), 665 ($M^+$-Cl, 25), 629 ($M^+$-2Cl, 13)

**3-succinimidobenzoic acid (23)**

0.36 mol 3-aminobenzoic acid (50 g) were dissolved in 550 ml THF by heating at 50 °C and 0.43 mol succinic anhydride (43 g) were added in ten portions. After stirring for 2 h at room temperature the resulting precipitate was filtered off and washed with a small amount of THF. The crude product was then suspended in 100 ml THF and treated with 1.44 mol acetic anhydride (147 g) and 0.36 mol sodium acetate (30 g) under reflux until an almost clear solution was formed. Excess acetic anhydride was hydrolysed by adding water and heating the solution under reflux for 1 h. After cooling to room temperature, concentrated hydrochloric acid was added until **23** began to precipitate. The suspension was cooled to 4 °C for 5 h. Then the precipitate was filtered off, washed with water and dried in vacuo to yield 71 g (0.32 mol, 88.8%) **23**.

$C_{11}H_9NO_4$ (219 g/mol), m.p.: 225 °C, calc.: C: 60.27%, H: 4.11%, N: 6.39%, found: C: 60.41%, H: 4.10%, N: 6.56%, $^1$H NMR (300 MHz, DMSO-$d_6$): δ = 13.1 (s, 1H, COOH), 7.9 (m, 2H, arom. H), 7.5 (m, 2H, arom. H), 2.7 (s, 4H, $H_2C$-$CH_2$), $^{13}$C NMR (75.4 MHz, DMSO-$d_6$): δ = 176.8 (OC-$CH_2CH_2$-CO), 166.5 (COOH), 132.9/131.4/131.3/129.1/128.8/128.0 (arom. C), 28.4 ($H_2C$-$CH_2$)

**3-succinimidobenzoyl chloride (24)**

0.21 mol **23** (46 g) were suspended in 200 ml of toluene and treated with 0.63 mol thionyl chloride (75 g) under reflux until a solution was formed. Excess thionyl chloride was removed by distillation and toluene was subsequently evaporated in vacuo. The brownish residue was crystallised from acetone and washed with small amounts of n-hexane. The product was dried in vacuo to yield 38.9 g (0.16 mol, 76.2%) of **24**.

$C_{11}H_8NO_3Cl$ (237.45 g/mol), m.p.: 100 °C, calc.: C: 55.59%, H: 3.37%, N: 5.90%, Cl: 14.93%, found: C: 55.90%, H: 3.19%, N: 5.73%, Cl: 14.62%, $^1$H NMR (300 MHz, DMSO-$d_6$): δ = 7.9 (m, 2H, arom. H),

7.5 (m, 2H, arom. H), 2.8 (s, 4H, $H_2C$-$CH_2$), $^{13}C$ NMR (75.4 MHz, DMSO-$d_6$): δ = 176.4 (OC-$CH_2CH_2$-CO), 166.0 (COCl), 132.5/131.7/131.0/129.6/128.4/127.5 (arom. C), 28.5 ($H_2C$-$CH_2$)

**1-(3-succinimidobenzoyl)-2-(*tert.*-butoxycarbonyl)-hydrazine (25)**

42.1 mmol **24** (10 g) and 42.1 mmol *tert.*-butylcarbazate (5.56 g) were dissolved in 160 ml THF and treated under vigorous stirring with 42.1 mmol triethylamine (5.85 ml). After 10 minutes the reaction was complete. The precipitated triethylammonium chloride was filtered off and the filtrate evaporated to dryness in vacuo, dissolved in ethyl acetate and washed twice with 100 ml 0.5 N hydrochloric acid and then twice with 100 ml saturated NaCl solution. The organic phase was concentrated and cooled to 0°C to yield 11.95 g (35.9 mmol, 85.3%) of **25** as a colourless powder.

$C_{16}H_{19}N_3O_5$ (333 g/mol), m.p.: 184 °C, calc.: C: 57.66%, H: 5.71%, N: 12.61%, found: C: 58.20%, H: 5.94%, N: 12.66%, $^1H$ NMR (300 MHz, DMSO-$d_6$): δ = 10.3/8.9 (2s, 2H, 2 NH), 7.9-7.4 (m, 4H, arom. H), 2.8 (s, 4H, $H_2C$-$CH_2$), 1.4 (s, 9H, 3 $CH_3$), $^{13}C$ NMR (75.4 MHz, DMSO-$d_6$): δ = 176.7 (OC-$CH_2CH_2$-CO), 165.1 (Ar-CONH), 155.3 (NCOO), 133.2/132.9/130.3/128.9/126.5/126.4 (arom. C), 79.2 ($C(CH_3)_3$), 28.4 (3 $CH_3$), 28.0 ($H_2C$-$CH_2$)

**3-succinimidobenzoylhydrazide trifluoroacetate (26)**

15.0 mmol **25** (5 g) were treated with 20 ml trifluoroacetic acid at room temperature, until a solution was formed. 100 ml diethyl ether were added under vigorous stirring resulting in the precipitation of **26**, which was separated by filtration and washed twice with 25 ml diethyl ether. The product was dried in vacuo to yield 4.16 g (11.99 mmol, 79.9%) of **26** as a colourless powder.

$C_{13}H_{12}N_3O_5F_3$ (347 g/mol), m.p.: 89 °C, calc.: C: 44.96%, H: 3.46%, N: 12.10%, found: C: 44.12%, H: 3.40%, N: 11.81%, $^1H$ NMR (300 MHz, DMSO-$d_6$): δ = 9.3 (bs, 4H, NH/$NH_3^+$), 7.9-7.6 (m, 4H, arom. H), 2.8 (s, 4H, $H_2C$-$CH_2$), $^{13}C$ NMR (75.4 MHz, DMSO-$d_6$): δ = 177.0 (OC-$CH_2CH_2$-CO), 165.4 (Ar-CONH), 162.1 (q, $CF_3COOH$, J = 33.7 Hz), 136.7/134.9/134.7/132.8/130.4/130.0 (arom. C), 121.9 (q, $CF_3COOH$, J = 295.4 Hz), 28.7 ($H_2C$-$CH_2$)

**4-[N,N'-bis-(*tert.*-butoxycarbonyl)-D,L-2,3-diaminopropanoyloxy]-benzaldehyde 3-succinimido-benzoylhydrazone (27)**

5.76 mmol **26** (2 g) and 5.76 mmol **15** (2.35 g) were suspended in 50 ml ethyl acetate and heated at 50 °C until precipitation occurred. The precipitate was filtered off and crystallised from methanol to yield 2.29 g (3.68 mmol, 63.9%) **27**.

$C_{31}H_{37}N_5O_9$ (623 g/mol), m.p.: 184 °C, calc.: C: 59.71%, H: 5.94%, N: 11.24%, found: C 59.51%, H: 6.13%, N: 11.03%, $^1H$ NMR (300 MHz, DMSO-$d_6$): δ = 11.9 (s, 1H, C=NNH), 8.5 (s, 1H, N=CH), 8.0-7.2 (m, 8H, arom. H), 7.4 (d, 1H, CHNH), 7.0 (t, 1H, $CH_2NH$), 4.3 (m, 1H, CH), 3.4 (m, 2H, $CH_2$), 2.8 (s, 4H, $H_2C$-$CH_2$), 1.4/1.3 (2s, 18H, 6 $CH_3$), $^{13}C$ NMR (75.4 MHz, DMSO-$d_6$): δ = 177.7 (OC-$CH_2CH_2$-CO), 170.5 (CHCO), 163.1 (C=NNH-CO), 156.5/156.2 (2 NCOO), 152.6 (C=N), 148.1/135.0/134.0/133.0/131.3/129.9/129.1/127.9/127.6/123.0 (arom. C), 79.6/79.1 (2 $C(CH_3)_3$), 55.1 (CH), 41.7 ($CH_2$), 28.4 ($H_2C$-$CH_2$), 28.0 (6 $CH_3$)

**4-[N,N'-bis-(*tert.*-butoxycarbonyl)-D,L-2,3-diaminopropanoyloxy]-acetophenone 3-succinimido-benzoylhydrazone (28)**

5.76 mmol **26** (2 g) and 5.76 mmol **16** (2.43 g) were dissolved in 50 ml THF and stirred for 2 h at room temperature. THF was then evaporated in vacuo and the residue dissolved in a minimal amount of ethyl acetate and purified by column chromatography (silica gel, ethyl acetate / n-hexane 1:1) to yield 1.20 g (1.89 mmol, 32.8%) **28**.

$R_f$ = 0.23 (ethyl acetate / n-hexane 1:1), $C_{32}H_{39}N_5O_9$ (637 g/mol), m.p.: 168 °C, calc.: C: 60.28%, H: 6.12%, N: 10.99%, found: C: 60.40%, H: 6.15 %, N: 10.70%, $^1H$ NMR (300 MHz, DMSO-$d_6$): δ = 10.9 (s, 1H, C=NNH), 7.9-7.0 (m, 8H, arom. H), 7.4 (d, 1H, CHNH), 6.9 (t, 1H, $CH_2NH$), 4.3 (m, 1H, CH), 3.4 (m, 2H, $CH_2$), 2.8 (s, 4H, $H_2C$-$CH_2$), 2.3 (s, 3H, N=C-$CH_3$), 1.4/1.3 (2s, 18H, 6 $CH_3$), $^{13}C$ NMR (75.4 MHz, DMSO-$d_6$): δ = 177.0 (OC-$CH_2CH_2$-CO), 169.9 (CHCO), 163.3 (C=NNH-CO), 155.8/155.6 (2 NCOO), 151.6 (C=N), 135.9/135.0/133.1/132.9/130.3/128.9/127.8/127.1/121.7 (arom. C), 78.9/78.4 (2 $C(CH_3)_3$), 54.4 (CH), 41.1 ($CH_2$), 28.7 ($H_2C$-$CH_2$), 28.3 (6 $CH_3$), 26.6 (N=C-$CH_3$)

### 5. Literature

[1] Heim, M. E. in: *Metal Complexes in Cancer Chemotherapy* (ed. B. K. Keppler), Verlag Chemie, Weinheim (1993), p. 9

[2] Maeda, H. and Matsumura, Y. *Crit. Rev. Ther. Drug Carrier Sys.* **6** (1989), 1930

[3] Kratz, F. and Beyer, U. *Drug Delivery* **5** (1998), 1

[4] Kratz, F., Beyer, U., Roth, T., Tarasova, N., Collery, P., Lechenault, F., Cazabat, A., Schumacher, P., Unger, C. and Falken, U. *J. Pharm. Sci.* **87** (1998), 338

[5] Kratz, F., Beyer, U., Collery, P., Lechenault, F., Cazabat, A., Schumacher, P., Falken, U. and Unger, C. *Biol. Pharm. Bull.* **21** (1998), 56

[6] Kratz, F., Beyer, U., Schumacher, P., Krüger, M., Zahn, H., Roth, T., Fiebig, H. H. and Unger, C. *Bioorg. Med. Chem. Lett.* **7** (1997), 617

[7] Beyer, U., Roth, T., Schumacher, P., Maier, G., Unold, A., Frahm, A. W., Fiebig, H. H., Unger, C. and Kratz, F. *J. Med. Chem.* **41** (1998), 2701

[8] Kratz, F., Beyer, U., Roth, T., Schütte, M. T., Unold, A., Fiebig, H. H. and Unger, C. *Arch. Pharm. Pharm. Med. Chem.* **331** (1998), 47

[9] Drevs, J., Hofmann, I., Marmé, D., Unger, C. and Kratz, F. *Drug Delivery* **6** (1999), 1

[10] Kratz, F., Beyer, U. and Schütte, M. T. *Crit. Rev. Ther. Drug Carrier Sys.* **16** (1999), 245

[11] Schütte, M. T., Schumacher, P., Unger, C., Mülhaupt, R. and Kratz, F. *Inorg. Chim. Acta* **267** (1998), 133

[12] Beyer, U., Krüger, M., Schumacher, P., Unger, C. and Kratz, F. *Monatshefte der Chemie* **128** (1997), 91

[13] Benassi, R. and Taddei F. *J. Chem. Soc., Perkin Trans. II* **10** (1985), 1629

[14] Slotta, K. H. and Kethur, R. *Chem. Ber.* **71** (1938), 335

**Received: November 29, 1999 - Accepted: December 17, 1999 - Received in revised camera-ready format: March 8, 2000**

